# Modern Approach to Ureteral Stones

**DOI:** 10.1100/tsw.2003.71

**Published:** 2003-09-03

**Authors:** Geert G. Tailly

**Affiliations:** Department of Urology, AZ KLINA, Augustijnslei 100, 2930 Brasschaat, Belgium

**Keywords:** ureteral stones, urolithiasis, urinary calculi, management of ureteral stones, management of urolithiasis, management of urinary calculi

## Abstract

Urolithiasis is a very common affliction of mankind. In western countries incidence is increasing steadily. An increasing proportion of patients are presenting with ureteral stones, of which renal colic most often is the first complaint and the most common reason for an emergency visit to a urologist. Proper imaging strategy is of paramount importance in the diagnosis of acute flank pain and in the subsequent therapy planning once a ureteral stone is diagnosed. Renal colic during pregnancy poses specific problems, both in imaging and therapy. Apart from the adequate treatment of renal colic, modern therapy of those ureteral calculi that will not pass spontaneously will consist of a judicious combination of ESWL (extracorporeal shock wave lithotripsy), endourology, and laparoscopy. Open surgery should only be reserved for limited and very specific indications. Although beyond the scope of this article, metaphylaxis should take an important role in the follow-up of stone patients in general.

### INTRODUCTION

Already present in ancient times[1,2,3] urolithiasis is a very common affliction of mankind. In Europe, the yearly incidence rate is about 0.5%, prevalence 4–10%, and the recurrence rate up to 80%[4]. Most stone centers currently identify 35–60% of their stone patients as suffering from ureteral stones.

Ureteral colic most often is the primary manifestation of the presence of a ureteral stone and accounts for most of the emergency visits to the urologist. Various degrees of obstruction of the collecting system, obstructive pyelonephritis and urosepsis, and deterioration of renal function may further complicate the problem. This review article endeavours to give an account of the modern approach to ureteral stones.

## IMAGING

### Imaging in Renal Colic

#### *Plain Abdominal Film of Kidneys, Ureters, and Bladder (KUB)[4,5,6,7]*

In the emergency work-up of patients presenting with renal colic a plain abdominal film or KUB usually is performed. A KUB will demonstrate most radio-opaque stones at all levels of the urinary tract; a KUB will give an idea of location and size of radio-opaque lithiasis but cannot indicate the degree of an eventual obstruction.

The interpretation of the KUB is further limited in patients with:

- Radiolucent stones
- Very small stones
- Stones overlying bony structures
- Calcified opacities outside the collecting system (phleboliths, calcified lymph nodes, etc.)

Also, a KUB should not be performed in pregnancy.

#### *Ultrasonography (US)[4,5,7,8,9,10]*

In the work-up of a patient with renal colic, US can detect:

- Location and size of a stone, radiolucent or radio-opaque, in the kidney, the very proximal and the very distal ureter
- Degree of obstruction

Further advantages are:

- No need for IV injection of contrast medium
- No exposure to radiation
- No side effects
- Safe in pregnancy

US cannot detect most ureteral stones, however. Especially in the management of pregnant women and children[8] with ureteral calculi, US plays an important role. In the detection of ureteral calculi, the sensitivity of unenhanced spiral CT-scan however is higher than the sensitivity of US[9,10,24,51) (Table 1).

**TABLE 1**
**Sensitivity and Specificity of KUB, US, IVU, and UHCT in the Detection of Ureteral Calculi[9,10,24,25,51]**

| | Sensitivity (%) | Specificity (%) |
|---|---|---|
| KUB | 44–77 | 80–87 |
| US | 52–73 | 100 |
| IVU | 87 | >94 |
| UHCT | 95–98 | 96–100 |

### *Intravenous Urography (IVU)[4,5]*

IVU has long been the imaging modality of choice in the assessment of patients presenting with renal colic. An IVU will give the following valuable information:

- Location and size of radio-opaque ureteral calculi
- Possible location of radiolucent calculi as a filling defect in the contrast medium. Cave differential diagnosis with other filling defects such as tumors of the urothelium
- Degree of obstruction
- Anatomical abnormalities of the collecting system
- Precise architecture of the collecting system: infundibulopelvic angle and length, width of the lower pole calyx, etc.

The following drawbacks need to be stressed[11]:

- Use of IV contrast medium: possible allergy to iodized contrast media
- Exposure to radiation
- Sometimes time consuming
- Cannot be used in patients with allergy to iodized media, patients with serum creatinine level >200 µmol/l, patients on Metformin, patients with myelomatosis, patients who are pregnant

### *Retrograde and Antegrade Pyelography[4,11]*

In selected cases these more invasive examinations can give valuable information in the assessment of a silent kidney in selected cases.

### *Computed Tomography (CT-Scan)[4,5,6,8,9,10,11,12]*

An unenhanced abdominal and pelvic CT-scan (UHCT) increasingly is considered the imaging modality of choice in the evaluation of patients with flank pain. Advantages are:

- No need for IV contrast medium
- Limited radiation exposure
- Less time consuming than IVU
- Diagnosis of other (nonurologic) causes of flank pain
- Diagnosis of concommitant pathology in other (nonurologic) systems
- High cost effectiveness

Meagher et al.[12] evaluated low-dose CT in suspected renal colic. With a comparable detection rate of 93% for ureteral calculi, mean radiation dose was 1.5 mSv for IVU and 3.5 mSv for unenhanced spiral CT. The mean examination time was 5 min for CT and 80 min for IVU[12].

Wong et al.[13] found unenhanced spiral CT-scan more effective than IVU in the detection of ureteral calculi and equally effective as IVU in the detection of urinary obstruction.

In a group of young patients aged 8–18 years, Lumerman et al.[14] found unenhanced spiral CT highly sensitive and specific in the detection of renal and ureteral calculi. Apart from that, unenhanced spiral CT-scan also proved useful in the detection of alternate pathology.

In a study comparing the expense of both CT and IVU in the evaluation of emergency cases with urolithiasis, Kennedy et al.[15] found CT significantly more expensive for the patient than IVU ($736 for CT vs. $304 for IVU). According to Dalla Palma et al.[24] however, the real costs of IVU (€80.72) and UHCT (€74) are comparable. The fact that CT is less time consuming, offers diagnosis of nonurological pathology, and does not carry the risks of the IV injection of contrast medium, however, may well make CT more cost effective than IVU.

Gallagher and Tolley[16] acknowledge the superiority of CT in the diagnosis of acute flank pain, but still consider IVU essential in the evaluation of the spatial anatomy of the pyelocalyceal system. This proves especially true in the determination of the treatment strategy for lower calyceal stones.

In a recent article, Heidenreich et al.[25] predicts, however, that UHCT will become the imaging procedure of choice in the evaluation of a patient with acute flank pain and presents excellent algorythms for its diagnosis.

- High sensitivity and specificity in the detection of ureteral stones
- Detection of extraurinary pathology
- No need for IV contrast medium
- Less time consuming than IVU

### Magnetic Resonance Imaging

Magnetic Resonance Urography (MRU) may be of value in diagnosing urinary obstruction by calculi especially during pregnancy[17]. Radiologic diagnosis of urinary lithiasis during pregnancy is complicated by a number of factors[17,18]:

- Pregnancy dilatation of the urinary tract:
  - Right side more affected than left
  - Starts at the end of the first trimester and progresses with the duration of gestation
  - Caused by
    - Hormonal factors: smooth muscle relaxation by high progesterone levels
    - Mechanical factors: compression of the distal ureters by the gravid uterus at the cross-over with the iliac vessels
- Risk of radiation to the fetus:
  - Highest risk during organogenesis (0–8 weeks after conception)
  - Dosage related: lower in well-planned diagnostic studies
- IV administration of iodized contrast medium
  - May cause adverse reaction to the mother
  - Adverse effects to fetus?

Hence, in pregnancy US is considered the imaging tool of choice in the diagnosis of ureteral obstruction. If US fails to solve the problem, MRU can be safely performed. Although MRU is considered a safe imaging procedure with no known teratogenic effects[17,19,20] it is suggested to use it with caution in the first trimester of pregnancy[21,22,23].

### Conclusions

According to Dalla Palma et al.[24], UHCT is the imaging procedure of choice in the evaluation of a patient with renal colic:

- Identification of the stone
- Evaluation of the degree of obstruction
- Detection of extraurinary abnormalities

Apart from above-mentioned advantages of UHCT, the following factors should be considered as well:

- The real cost of UHCT is not or only slightly higher than the cost of IVU[24].
- The radiation dose delivered by a judiciously executed UHCT is only slightly higher than by an IVU.

If UHCT is not readily available a plain film KUB in combination with US can be considered the method of choice. In unresolved cases, IVU should be added.

In pregnancy, US is considered the method of choice, complemented if necessary by MRU[17,19]. Even a limited-dose IVU should be discouraged during pregnancy[19]. Because of the high radiation dose, UHCT is contraindicated during the entire duration of the pregnancy.

### Imaging in *In Situ* ESWL of Ureteral Stones

Treatment strategies in ESWL are to a great extent influenced by the imaging system available in a lithotriptor. Early lithotriptors were equipped either with ultrasound or fluoroscopy. In order to meet all the challenges in modern integrated stone management, present day lithotriptors ideally should be equipped with both imaging modalities. Table 2 displays the advantages and disadvantages of ultrasonic and fluoroscopic targeting in ESWL while Table 3 displays advantages and disadvantages of an in-line or an out-line scanner in ultrasonic targeting in ESWL[26].

**TABLE 2**
**Advantages and Disadvantages of Ultrasonic and Fluoroscopic Targeting in ESWL**

| Fluoroscopy | Ultrasound |
|---|---|
| Advantages | |
| *In situ* treatment of ureteral stones in all parts of the ureter | Easy targeting of radiolucent stones |
| Shorter learning curve | Easier targeting of smaller renal stones |
| | Real-time image: easier and faster adaptation of focusing |
| | No exposure to radiation |
| Disadvantages | |
| No direct targeting of radiolucent stones | *In situ* treatment of ureteral stones is possible only for very proximal and very distal ureteral calculi |
| Small stones sometimes difficult to locate | Longer learning curve |
| No real-time image | |
| Exposure to radiation | |

**TABLE 3**
**Advantages and Disadvantages of the Use of an In-Line or Out-Line Scanner in Ultrasonic Targeting in ESWL**

| Ultrasound | |
|---|---|
| **In-Line Scanner** | **Out-Line Scanner** |
| Advantages | |
| Easier dissociation between multiple stones | For kidney stones, the most appropriate window can be chosen: avoid ribshadows |
| Easier targeting of very proximal and very distal ureteral stones | Better appraisal of fragmentation |
| In the shock wave path | Use as diagnostic scanner |
| | Can be exchanged for 5 MHz scanner: children |
| | Far better image quality |
| Disadvantages | |
| Ribshadows may hide stones from view | Very proximal ureteral stones sometimes more difficult to find |
| Poorer image quality | More difficult patient positioning for prevesical stones |
| Negative effect on shock wave power | |
| Possible damage of scanner by SW | |

### TREATMENT

#### Renal Colic

Renal colic is an excruciating form of pain. Mild renal colic can be treated on an ambulatory basis. Severe and/or repeated renal colic mandates hospitalization. Mainstays in the conservative treatment of renal colic are:

- A warm bath will result in muscle relaxation
- Limit fluid intake
- Drug therapy: NSAID or narcotic analgesics

When medical treatment does not result in pain relief, relief of obstruction may be necessary.

#### Spontaneous Passage

Given time, most ureteral stones will pass spontaneously. According to Hesse et al.[4], 60–70% will do so. According to the Guidelines on Urolithiasis of the EAU[11], 25% of proximal ureteral stones, 45% of mid-ureteral stones, and 70% of distal ureteral stones pass spontaneously. According to the Report on The Management of Ureteral Calculi of the AUA[29], 98% of stones smaller than 5 mm in diameter, especially in the distal ureter, will pass spontaneously.

The following factors will determine the time that both patient and urologist are prepared or allowed to wait for spontaneous passage:

- Location and size of the stone
- Severity of renal colic
- Presence of urinary infection
- Degree of obstruction
- Renal function
- Special conditions: solitary kidney, transplant kidney, pregnancy, etc.

- Professional activity of the patient

In general, in the era prior to endourology and ESWL more patience was wise, as the alternative most often was open surgery. Following the introduction of minimally invasive techniques, the active treatment of ureteral lithiasis can be more "aggressive" as the minimally invasive treatment modalities can solve the problem in a more patient-friendly, more expedient way with fewer complications thus greatly reducing overall morbidity caused by a ureteral stone.

### Endourology/ESWL

Endourology is defined as *"the closed, controlled manipulation of the entire urinary tract"*. Since its introduction in 1980[27], ESWL has become the treatment of choice for virtually all calculi in the urinary tract regardless of their location[4,11,26,27,28,29]. Given the proper imaging modality and positioning technique, any ureteral stone can be targeted for ESWL on a modern lithotriptor[28]. Together with ESWL, endourology (and especially retrograde ureterorenoscopy [URS]) can be considered first line treatment of any ureteral calculus[11,29].

In which instances ESWL or URS should be preferred in the primary treatment of ureteral calculi is a matter of ongoing debate[31,32,33,34,35,36,37,38,39]. In the management of ureteral stones, ESWL generally is considered the treatment of first choice[32,34,36], as it is the minimally invasive technique "par excellence" and also achieves excellent stone free rates. The drawback is that multiple treatments may be necessary and salvage URS may be required in cases were ESWL fails. Therefore some authors advocate URS as the first choice treatment, especially as instruments have become more sophisticated and URS can be performed in an ambulatory setting with a minimum of complications[37,38]. Primarily due to the investment costs of a lithotriptor, URS can be considered more cost effective than ESWL[39].

The choice between ESWL or URS as first line treatment for a ureteral calculus will therefore have to be made on the basis of several considerations:

- Availability and type of lithotriptor
- Type, size, and location of the stone
- Presence of UTI
- Endourologic equipment and skill
- Experience with ESWL and/or URS
- Patient's preference
- Cost

Table 4 displays a schematic overview of the management of ureteral calculi according to the Guidelines on Urolithiasis of the EAU[11]. Table 5 gives a schematic overview of the recommendations of the Ureteral Stones Clinical Guidelines Panel of the AUA[29]. An algorithm for the management of ureteral calculi that has been in use for many years in our stone center[28] is dispayed in Fig. 1.

Table 6 displays the recommendations of the EAU for the treatment of Steinstraβe[11]. In Fig. 2, an algorithm for the management of post-ESWL obstruction in use in our stone center is displayed[30].

**TABLE 4**
**Management of Ureteral Calculi: Guidelines on Urolithiasis of the EAU[11]**

| | Proximal Ureter (All Sizes) | Mid-Ureter (All Sizes) | Distal Ureter (All Sizes) |
|---|---|---|---|

| | | | |
|---|---|---|---|
| Radio-opaque stones | ESWL *in situ*<br>“Push and bang”<br>PNL + antegrade URS<br>Retrograde URS + contact disintegration | ESWL *in situ*<br>Retrograde URS + contact disintegration<br>Ureteral catheter or IV contrast + ESWL<br>“Push and bang”<br>PNL + antegrade URS | ESWL *in situ*<br>Retrograde URS + contact disintegration<br>Ureteral catheter + ESWL |
| Infection stones/stones with infection | Strategies cfr. radio-opaque stones/antibiotic coverage | Strategies cfr. radio-opaque stones/antibiotic coverage | Strategies cfr. radio-opaque stones/antibiotic coverage |
| Uric acid/urate stones | Stent + oral chemolysis<br>ESWL *in situ* + oral chemolysis<br>PNL + antegrade URS<br>Retrograde URS + contact disintegration | ESWL *in situ*<br>Retrograde URS + contact disintegration<br>Ureteral catheter or IV contrast + ESWL<br>“Push and bang”<br>Stent + oral chemolysis<br>PNL + antegrade URS | ESWL *in situ*<br>Retrograde URS + contact disintegration<br>Ureteral catheter + ESWL<br>Nephrostomy + antegrade contrast + ESWL *in situ* |
| Cystine stones | ESWL *in situ*<br>“Push and bang”<br>PNL + antegrade URS<br>Retrograde URS + contact disintegration | ESWL *in situ*<br>Retrograde URS + contact disintegration<br>Ureteral catheter + ESWL<br>“Push and bang”<br>PNL + antegrade URS | ESWL *in situ*<br>URS + contact disintegration<br>Ureteral catheter + ESWL |

In our opinion, the mere introduction of a ureteral catheter to improve visualization and targeting of a ureteral stone for ESWL[11] should be discouraged and replaced by outright retrograde URS of the targeted stone. Retrograde URS, in fact, is only slightly more invasive than the introduction of a ureteral catheter and can be considered appropriate and definitive treatment. Also, the subsequent ESWL after the introduction of a ureteral catheter to improve targeting may not offer a 100% success rate and a “salvage” URS may still be needed. Routine stenting prior to ESWL does not improve outcome[29] and thus should be discouraged.

As in ESWL, we are convinced that routine stenting in URS is not necessary. Uneventful URS does not profit from stenting and the patient may only experience the discomfort often associated with the presence of a ureteral stent: flank pain exacerbated during voiding, frequency and urgency, pain in the suprapubic and iliac fossa area, etc. Therefore, we reserve post-URS stenting to those URS that were difficult or complicated: narrow distal ureter with difficult access, long-term impaction of the ureteral stone treated, perforation of the ureter, etc.

**TABLE 5**
**Management of Ureteral Calculi: Report on the Management of Ureteral Calculi of the AUA[29]**

| | Proximal Ureter | | Distal Ureter | |
|---|---|---|---|---|
| | ≤1 cm | >1 cm | ≤1 cm | >1 cm |
| Standard | Open surgery ≠ first | | Open surgery ≠ first | Blind basketing is |

| | | | | |
|---|---|---|---|---|
| | line treatment | | line treatment | not recommended |
| Guideline | ESWL = first line treatment | Open surgery ≠ first line treatment | Blind basketing without fluoroscopy and guidewire cannot be encouraged | Open surgery ≠ first line treatment |
| Option | | ESWL = PNL = URS = acceptable treatment choices | ESWL = URS = acceptable treatment choices | ESWL = URS = acceptable treatment choices |
| Guideline | Routine stenting prior to ESWL is not recommended | | | |

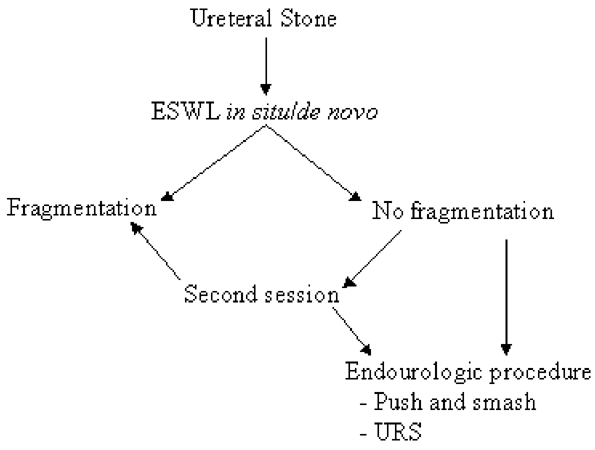

**FIGURE 1**. Algorithm in the treatment of ureteral stones[28].

### Laparoscopy

After rather a slow start, laparoscopic surgery is rapidly gaining ground in urology. Both the kidney and ureter can be approached via a transperitoneal[41] or retroperitoneoscopic[40,42] route.

Laparoscopic ureterolithotomy is considered safe and reliable[40] but at this moment in time, its indications remain limited to a salvage intervention following failure of URS and/or ESWL. In rare occasions of impacted ureteral stones in solitary kidneys and in well-trained hands, it can be considered the treatment of choice[40].

**TABLE 6**
**Recommendations for Treatment of Steinstraβe: Guidelines on Urolithiasis of the EAU[11]**

| Position | Unobstructed | Obstructed and/or Symptomatic |
|---|---|---|
| Proximal ureter | ESWL | PN (= percutaneous nephrostomy)<br>Stent<br>ESWL |
| Mid-ureter | ESWL | PN<br>Stent<br>ESWL |
| Distal ureter | ESWL<br>URS | PN<br>ESWL<br>URS |

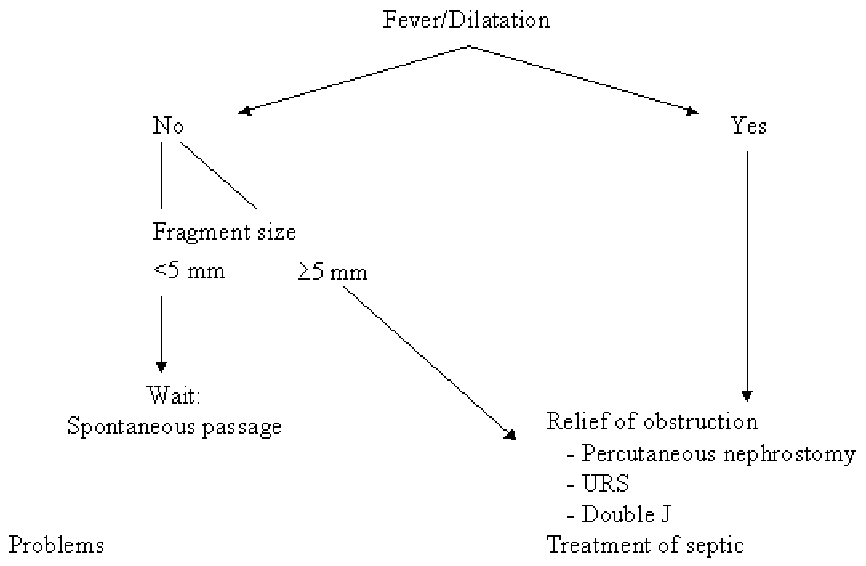

**FIGURE 2**. Algorithm for the management of post-ESWL obstruction[30].

### Open Surgery

Most ureteral calculi are best treated using URS and ESWL as minimally invasive techniques. When both fail, a laparoscopic approach should be preferred as a minimally invasive salvage technique. Therefore, indications for open surgery in the management of ureteral calculi have to be considered extremely rare. Advocates of minimally invasive techniques would not mind seeing open surgery becoming obsolete altogether in the treatment of ureteral (and renal) calculi.

### Pregnancy[43,44,45,46,47,48,49,50]

Basically, the management of ureteral calculi during pregnancy is conservative and consists of a relative reduction of fluid intake, rest, and analgetics (no NSAID during pregnancy). Active treatment is only indicated in the presence of colic refractory to conservative treatment, ureteral stone in a solitary kidney, (threat of) urosepsis, (threat of) premature labor that does not respond to tocolytic treatment. Active treatment should still be minimally invasive and should consist in temporary urinary diversion either by percutaneous nephrostomy or a double-J-catheter. Definitive treatment is best delayed until the postpartum. This conservative management is advocated in the Guidelines on Urolithiasis of the EAU[11].

In the event that active treatment other than temporary diversion is considered necessary, both ESWL and PNL are contraindicated: ESWL is contraindicated because of the potential disruptive effects of SW on the fetus; PNL is contraindicated because of patient positioning, potentially prolonged anesthesia, and the need for fluoroscopy. Ureteral calculi needing active treatment other than temporary diversion are therefore best managed by URS executed by a highly experienced endourologist[11,43].

Shokeir et al.[46] consider URS safe and reliable even in the last trimester of pregnancy and even for larger stones. All authors, however, advocate conservative managment reserving more invasive therapy only in the indications mentioned. All authors also agree that URS in pregnant women should not be undertaken by the less experienced.

### METAPHYLAXIS[4,11]

The prevention of recurrence of urolithiasis is an equally important aspect in the overall management of urinary calculi. A distinction can be made between “general metaphylactic measures” intended for all stone patients and “specific metaphylactic measures” to be reserved for the metaphylaxis of specific stone types in recurrent stone formers. The extensive discussion of metaphylaxis however is beyond the scope of this overview: good guidelines are offered by both Hesse et al.[4] and the Guidelines on Urolithiasis of the EAU[11].

### BIOSKETCH

**Geert G. Tailly, M.D.,** graduated with distinction from the University of Leuven Medical School in 1978. He spent 2 years (1978–1980) General Surgery Residency at the Imeldaziekenhuis Bonheiden and 4 years (1980–1984) Urology Residency at the University Clinics of the Catholic University of Leuven, Department of Urology (Prof. Dr. H. Verduyn and Prof. Dr. R.L. Vereecken).

From 1984–1994, Dr. Tailly was full-time Urologist at the Algemene Kliniek H. Familie, Gent, Belgium and during that time (1984–1986), was Ad Interim Urologist at the Algemeen Ziekenhuis St. Lucas, Brugge, Belgium. In 1992, he became a Fellow of the European Board of Urology. Since 1994, Dr. Tailly has been Staff Urologist at Klina, Antwerp and Chief of the Department since 1998. His main interests are endourology, ESWL, and minimally invasive techniques in urology.

Dr. Tailly has 79 publications, including posters and videos mostly on endourology and ESWL, in the following international journals: *British Journal of Urology, Journal of Endourology*, *The Journal of Urology*, *Archivos Espagnoles de Urologia,* and *TheScientificWorldJOURNAL*. He has reviewed articles for Journal of *Endourology, Journal of Urology, European Urology, Urology.*

He is a member of Belgische Vereniging voor Urologie; Endourological Society; European Association of Urology; Société Internationale d'Urologie (SIU) ; European Board of Urology (Fellow of the EBU); AUA: Corresponding Member; ISMST: International Society for MusculoSceletal Shockwave Therapy; Belgische Vereniging voor Musculosceletale Schokgolf Therapie (BVMST): Founding Member; New York Academy of Sciences; American Association for the Advancement of Science.
